# Quantitative trait locus analysis of resistance to panicle blast in the rice cultivar Miyazakimochi

**DOI:** 10.1186/s12284-014-0002-9

**Published:** 2014-04-10

**Authors:** Takeaki Ishihara, Yuriko Hayano-Saito, Shinichi Oide, Kaworu Ebana, Nghia Tuan La, Keiko Hayashi, Taketo Ashizawa, Fumihiko Suzuki, Shinzo Koizumi

**Affiliations:** 1 National Agricultural Research Center, National Agriculture and Food Research Organization, 3-1-1 Kannondai, Tsukuba 305-8666, Ibaraki, Japan; 2 Present address: Molecular Microbiology and Biotechnology group, Research Institute of Innovative Technology for the Earth, 9-2 Kizugawadai, Kizugawa 619-0292, Kyoto, Japan; 3 National Institute of Agrobiological Sciences, 2-1-2 Kannondai, Tsukuba 305-8602, Ibaraki, Japan; 4 National Plant Resources Center, An Khanh, Hoai Duc Hanoi, Vietnam; 5 Present address: Technical Adviser, Tsukuba International Center, Japan International Cooperation Agency, 3-6 Koyadai, Tsukuba 305-0074, Ibaraki, Japan

**Keywords:** Oryza sativa L, Magnaporthe oryzae, Panicle blast resistance, QTL

## Abstract

**Background:**

Rice blast is a destructive disease caused by *Magnaporthe oryzae*, and it has a large impact on rice production worldwide. Compared with leaf blast resistance, our understanding of panicle blast resistance is limited, with only one panicle blast resistance gene, *Pb1*, isolated so far. The *japonica* cultivar Miyazakimochi shows resistance to panicle blast, yet the genetic components accounting for this resistance remain to be determined.

**Results:**

In this study, we evaluated the panicle blast resistance of populations derived from a cross between Miyazakimochi and the Bikei 22 cultivar, which is susceptible to both leaf and panicle blast. The phenotypic analyses revealed no correlation between panicle blast resistance and leaf blast resistance. Quantitative trait locus (QTL) analysis of 158 recombinant inbred lines using 112 developed genome-wide and 35 previously reported polymerase chain reaction (PCR) markers revealed the presence of two QTLs conferring panicle blast resistance in Miyazakimochi: a major QTL, *qPbm11*, on chromosome 11; and a minor QTL, *qPbm9*, on chromosome 9. To clarify the contribution of these QTLs to panicle blast resistance, 24 lines homozygous for each QTL were selected from 2,818 progeny of a BC_2_F_7_ backcrossed population, and characterized for disease phenotypes. The panicle blast resistance of the lines harboring *qPbm11* was very similar to the resistant donor parental cultivar Miyazakimochi, whereas the contribution of *qPbm9* to the resistance was small. Genotyping of the BC_2_F_7_ individuals highlighted the overlap between the *qPbm11* region and a locus of the panicle blast resistance gene, *Pb1*. Reverse transcriptase PCR analysis revealed that the *Pb1* transcript was absent in the panicles of Miyazakimochi, demonstrating that *qPbm11* is a novel genetic component of panicle blast resistance.

**Conclusions:**

This study revealed that Miyazakimochi harbors a novel panicle blast resistance controlled mainly by the major QTL *qPbm11. qPbm11* is distinct from *Pb1* and could be a genetic source for breeding panicle blast resistance, and will improve understanding of the molecular basis of host resistance to panicle blast.

## Background

Rice blast is caused by the fungus pathogen *Magnaporthe oryzae*, and is one of the most destructive diseases of rice (*Oryza sativa*) worldwide. The disease pathosystem comprises two major interrelated phases: leaf blast and panicle blast, with reproduction of the blast fungus on leaves serving as an infective source for panicle blast (Ou [1985]). The fungus infects panicles as well as leaves, and prevents grain filling after heading (Katsube and Koshimizu [1970]). Many leaf blast resistant rice cultivars have been identified (Miah et al. [2013]), and introducing leaf blast resistance traits into rice cultivars is one strategy to reduce the incidence of rice blast on panicles.

In general, the use of resistance traits in crop cultivation is a promising and effective method for disease control. This is because resistance results in lower disease severity and reduces the need for chemical applications, thus reducing environmental impacts as well as production costs. Compared with leaf blast resistance, less is known about the genetic components for panicle blast resistance, which is indispensable for stable rice production. The development of genetic resources for rice panicle blast resistance has been limited due to the lack of a standardized method that evaluates panicle blast resistance using a stable artificial inoculation technique, either under an incubator or/and in greenhouse conditions. Presently, field assays under natural conditions are performed to evaluate panicle blast resistance once a year in Japan (Fujii and Hayano-Saito [2007]); these assays require replication over several years for precise assessment. In the field, disease severity is largely affected by weather conditions within the rice-growing period. Furthermore, variations in heading date among individual plants further complicate the evaluation of resistance levels. These technical problems are obstacles to the exploration of new gene resources and the progress of genetic analysis of rice panicle blast resistance.

In Japan, only a few rice cultivars harboring panicle blast resistance have been identified: Tsukinohikari (Koumura et al. [1985a]); Zyugoyamochi (Koumura et al. [1985b]); and Miyazakimochi (Uchiyamada et al. [1979])]. To date, *Pb1*, derived from the *indica* cultivar Modan, is the only known gene for panicle blast resistance. *Pb1* has been introduced into commercial rice cultivars in Japan (Fujii and Hayano-Saito [2007]), and cultivars have remained resistant to panicle blast for over 30 years, demonstrating the durability of *Pb1*-dependent resistance (Fujii et al. [2005]). Nevertheless, novel genetic resources for the stable application of panicle blast resistance are being considered in preparation for the emergence of new virulent *M. oryzae* races.

**Figure 1:**
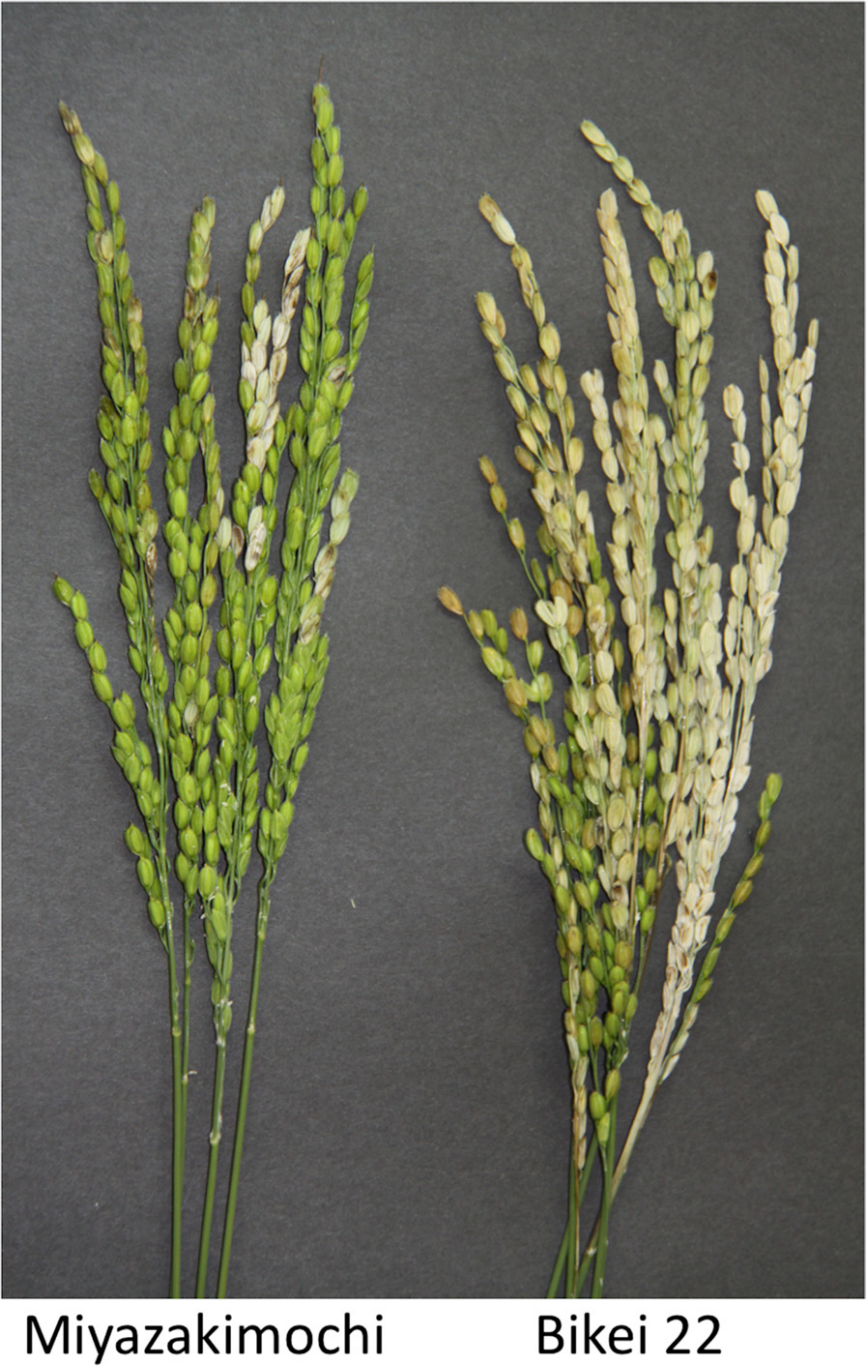
**Phenotypes of panicle blast in rice.** Phenotypes of panicle blast in Miyazakimochi (left) and Bikei 22 (right). The majority of white grains in the photograph are infected with blast. They most likely dried out because of diseased rachis-branches and spike necks.

The *japonica* rice cultivar Miyazakimochi shows resistance to panicle blast (Figure 1). Two preliminary quantitative trait locus (QTL) analyses using a population developed from a cross between Miyazakimochi and *japonica* rice cultivar Bikei 22, have been performed under different conditions to investigate blast disease (La et al. [2002]; Koizumi et al. [2005], both were abstracts of oral presentations). The first analysis of the phenotypes of 121 F_3_ lines and the genotypes of the F_2_ individuals with 51 simple sequence repeat (SSR) and 17 amplified fragment length polymorphism (AFLP) markers was performed in a experimental field with mild incidence of blast disease (La et al. [2002]). The second analysis, using the phenotypes of 126 lines out of the 171 F_6_ recombinant inbred lines (RILs) and the genotypes of the bulked F_6_ genomic DNAs with 41 SSR and 59 AFLP markers, was performed in a experimental field with high incidence of blast disease (Koizumi et al. [2005]). Both QTL analyses suggested the presence of a major QTL on rice chromosome 11, although no clear information concerning minor QTLs was proposed; there were no minor QTLs in the first analysis and one in the second.

**Figure 2:**
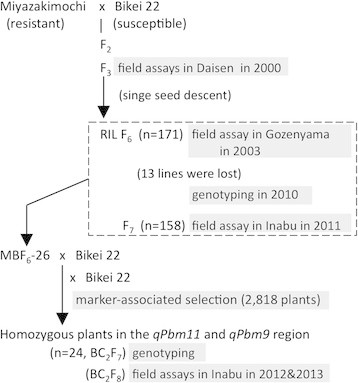
Flowchart showing the development of plant materials used in this study.

In this study, to confirm and resolve this issue, we developed genome-wide polymorphic DNA markers between Miyazakimochi and Bikei 22, and conducted a new QTL analysis under high-pressure condition for panicle blast disease, using the advanced generations of the same population in the above preliminary analyses (Figure 2). Furthermore, we examined the contribution of the identified QTLs for resistance to panicle blast and discuss their possible application in rice breeding programs.

## Results

### Characterization of resistance to panicle blast in Miyazakimochi

In the experimental paddy field, Miyazakimochi showed resistance to panicle blast, whereas panicles of the susceptible cultivar Bikei 22 were severely damaged (Figure 1). To characterize the panicle blast resistance of Miyazakimochi in more depth, we performed field assays using three generations developed from a cross of Miyazakimochi and Bikei 22. This was performed at three different experimental fields (Figure 2): F_3_ lines in Daisen (2000, Akita Prefecture); RILs at the F_6_ generation in Gozenyama (2003, Ibaraki Prefecture); and F_7_ RILs in Inabu (2011, Aichi Prefecture). In these fields, the pathogenic Japanese race (007.0), which is virulent to both Miyazakimochi and Bikei 22, is dominant, and local conditions are suitable for blast disease development.

The resistance score of Miyazakimochi ranged from 0.83 to 2.83 (average 1.83) in Daisen, and from 4.33 to 5.17 (average 4.68) in Gozenyama, but showed nearly susceptible scores, from 5.43 to 6.40 (average 5.82) in Inabu. Although the panicle blast severity scores of Miyazakimochi fluctuated at the three fields, the range in scores of Miyazakimochi never overlapped those of Bikei 22. The differences in average panicle blast severity scores between Miyazakimochi and Bikei 22 were statistically significant at *P* < 0.01 (Student's *t* test with Box-Cox transformation), clearly demonstrating the difference in panicle resistance between the two cultivars.

**Figure 3:**
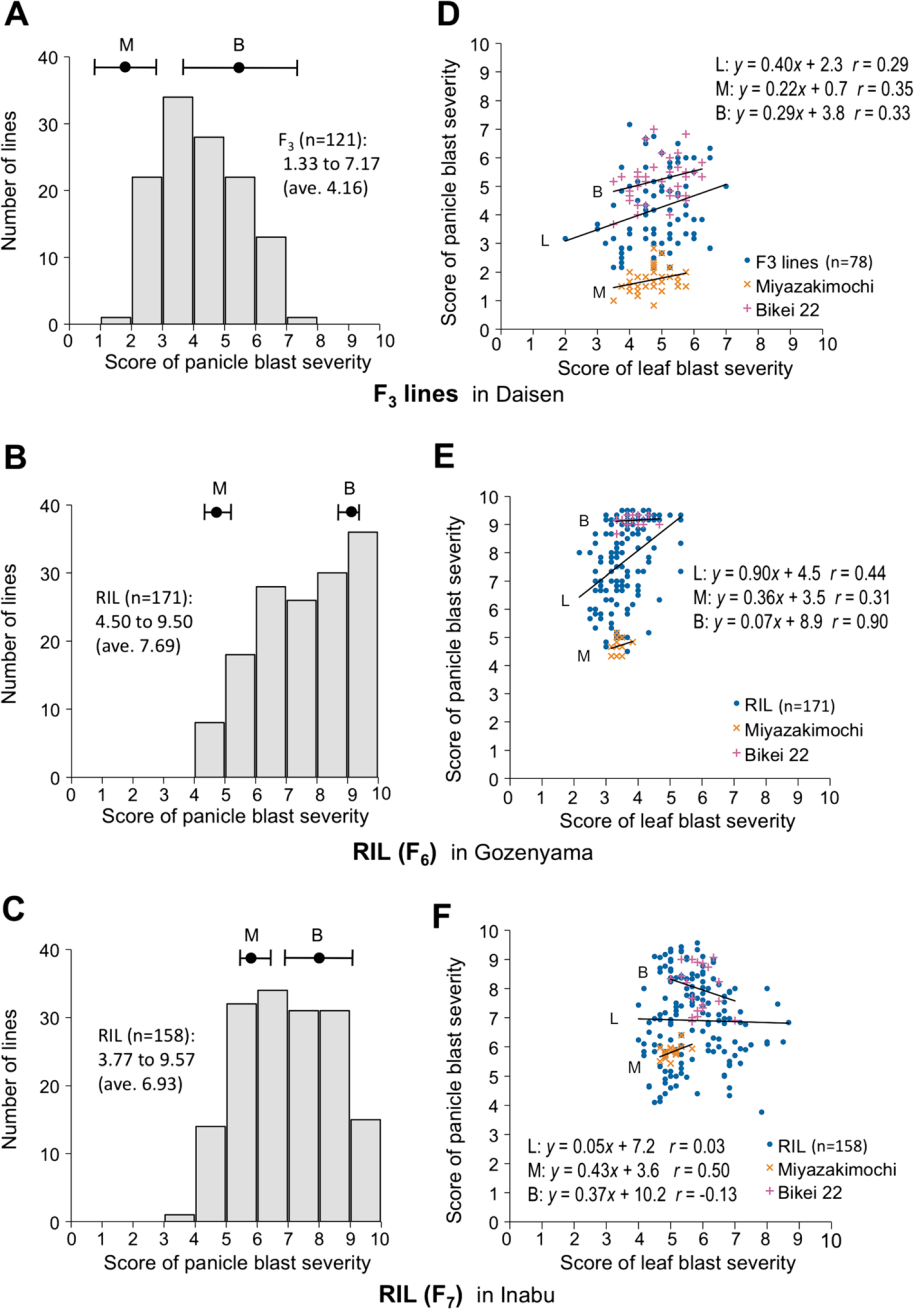
**Characterization of panicle blast severity distribution in progeny lines of Miyazakimochi/Bikei 22. (A–C)** Frequency distribution of the severity of panicle blast among progeny lines derived from a cross between Miyazakimochi (M) and Bikei 22 (B). Closed circles and error bars, at the top of the graph indicate the average and range of panicle blast severity of parental cultivars, respectively. **(D–F)** Correlation between panicle blast and leaf blast severity. Regression lines, equations, and correlation coefficients (*r*) are shown for the progeny lines (L) and the parental cultivars (M and B).

The frequency distributions of panicle blast severity in the 121 F_3_ lines, 171 F_6_ RILs, and 158 F_7_ RILs were asymmetric and continuous (Figure 3A, B, and C). The distribution of F_6_ and F_7_ RILs in the high-pressure fields of rice blast at Gozenyama and Inabu shifted to the susceptible side, while the F_3_ lines in the mid-pressure field at Daisen showed a slightly resistance-inclined distribution. The frequency distributions in the three tested generations under the different field conditions were not bimodal, suggesting that multiple loci are involved in the panicle blast resistance of Miyazakimochi.

To determine whether leaf blast resistance affects panicle blast in Miyazakimochi, we examined three generations of 78 F_3_ lines assayed in Daisen, 171 F_6_ RILs in Gozenyama, and 158 F_7_ RILs in Inabu for resistance to leaf and panicle blast (Figure 3D, E, and F). In terms of leaf blast severity, Miyazakimochi and Bikei 22 were almost indistinguishable in the three fields. The correlation coefficients between the panicle and leaf blast severity scores were approximately 0.29 in the F_3_ lines, 0.44 in the F_6_ RILs, and 0.03 in the F_7_ RILs, which implied a weak positive linear relationship between the leaf blast resistance and panicle blast resistance of Miyazakimochi.

### QTL analysis for panicle blast resistance

**Figure 4:**
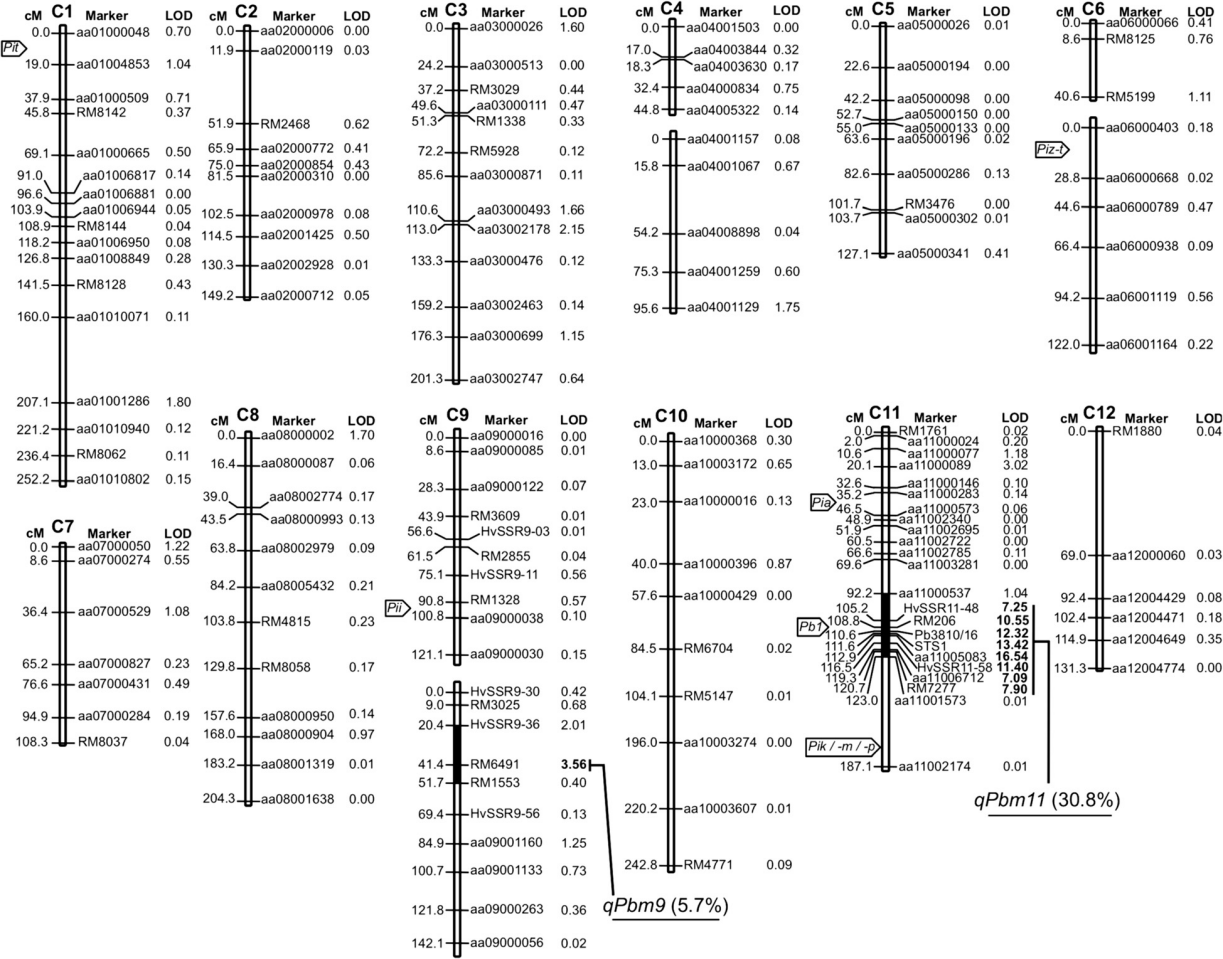
**Linkage map and the positions of QTLs for panicle blast resistance in Miyazakimochi.** The linkage map was constructed from 158 RILs derived from a cross between Miyazakimochi and Bikei 22. LOD scores in bold represent those above the 3.5 threshold. Black shading indicates the locations of the putative QTLs *qPbm9* and *qPbm11*. Contribution proportions are in parentheses. The locations of the major isolated resistance genes *Pia* (Okuyama et al. [2011]), *Pii* (Takagi et al. [2013]), *Pik* (Zhai et al. [2011]), *Pik-m* (Ashikawa et al. [2008]), *Pik-p* (Yuan et al. [2011]), *Piz-t* (Zhou et al. [2006]), *Pit* (Hayashi et al. [2010a]), and *Pb1* (Hayashi et al. [2010b]) are noted.

A set of 158 F_6_ RILs was used for QTL analysis. F_6_ individuals were genotyped using 147 polymorphic markers between Miyazakimochi and Bikei 22; these included 112 newly developed markers (Additional file 1), and covered almost all of the 12 chromosomes. A linkage map was constructed using MAPMAKER/EXP (Figure 4). This covered 2169.8 cM on the 12 chromosomes, with an average distance between adjacent markers of 14.9 cM. QTL analysis was performed with Windows QTL Cartographer, using phenotypic data of F_7_ lines obtained from a field assay in Inabu in 2011. Two QTLs with logarithm of odds (LOD) scores over the threshold value of 3.5 (*P* < 0.05) were detected on the long arms of chromosomes 9 and 11 (Figure 4). A major QTL, designated *qPbm11*, was identified in the 30.8 cM region between markers aa11000537 and aa11001573 on chromosome 11, with a contribution of 30.8% to the phenotype. The peak of the QTL (LOD = 16.54) was positioned at aa11005083. The minor QTL (LOD = 3.56), designated *qPbm9*, was detected in the region of marker RM6491 on chromosome 9, with a contribution of 5.7% to the phenotype.

### *Contribution of* qPbm11 *and* qPbm9 *to panicle blast resistance*

**Figure 5:**
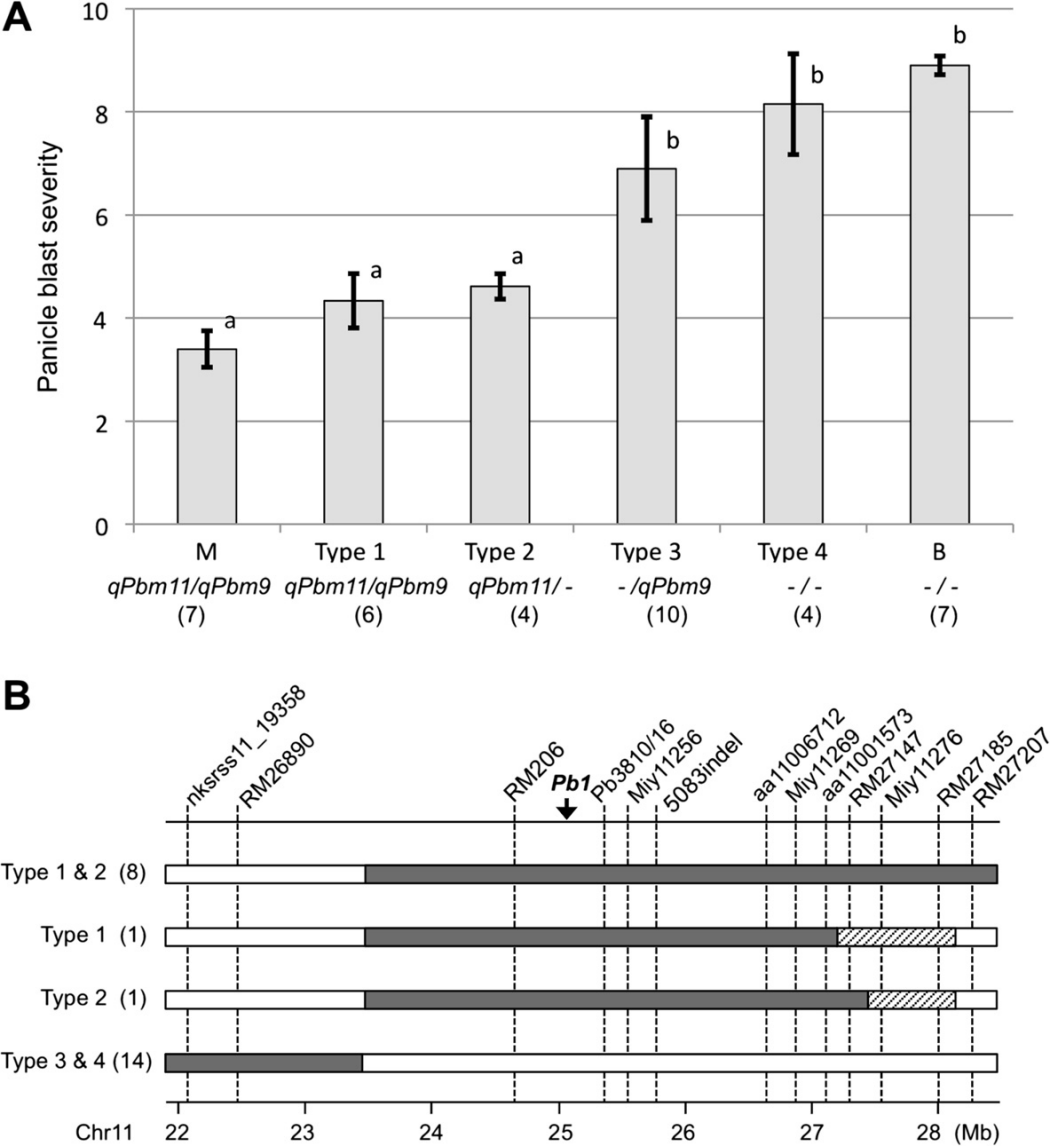
**Major contribution of*qPbm11*for panicle blast resistance and its location. (A)** Effect of two QTLs on panicle blast resistance. M and B represent the parental cultivars of Miyazakimochi and Bikei 22, respectively. QTLs in each group are indicated under group names *qPbm11/qPbm9*, *qPbm11/-, −/qPbm9*, and *−/−.* Bars are mean ± standard deviation of the tested number in parentheses. Bars with the same letter do not differ significantly according to the Tukey-Kramer test with Box-Cox transformation (*P* < 0.01). **(B)** Genotypes of the BC_2_F_7_ individuals in Types 1 to 4. Black and white bars indicate homozygous regions derived from Miyazakimochi and Bikei 22, respectively. The hatched bar is the heterozygous interval. Vertical lines represent marker positions with reference to the genomic sequence of Nipponbare (build 5, http://rapdblegacy.dna.affrc.go.jp/download/index.html). A black arrow indicates the position corresponding to *Pb1* isolated by Hayashi et al. ([2010b]). The number of lines showing the same genotype is given in parentheses after the group name.

To measure the combined contribution of *qPbm9* and *qPbm11* to panicle blast resistance, we screened BC_2_F_7_ individuals for those homozygous for the two QTL regions (Figure 2). The parental line MBF_6_-26 was Miyazakimochi-homozygous for both QTLs: between markers aa11000537 and aa11001573 on chromosome 11, and between markers aa09001133 and RM6491 on chromosome 9. Among the 2,818 BC_2_F_7_ individuals, 24 individuals were identified as homozygous for the Miyazakimochi or Bikei 22 genotype at the two QTL regions: between markers RM206 and RM27147, and between markers RM6491 and RM1553. We classified the progeny into four groups based on the genotypes of the QTL regions (Figure 5A): individuals harboring QTLs *qPbm11* and *qPbm9* (Type 1, *qPbm11/qPbm9*); individuals harboring *qPbm11* (Type 2, *qPbm11/-*); individuals harboring *qPbm9* (Type 3, −*/qPbm9*); and individuals lacking both QTLs (Type 4, *−/−*).

In the evaluation of panicle blast severity in the BC_2_F_8_ lines derived from each of the 24 BC_2_F_7_ individuals, a clear difference was observed between groups harboring *qPbm11* (Types 1 and 2) and those lacking *qPbm11* (Types 3 and 4). The average disease severity scores of Types 1 and 2 were significantly lower than those of Types 3 and 4 (*P* < 0.01). There was no significant difference at *P* < 0.01 between Type 1 and Type 2 and between Type 3 and Type 4, respectively. Nevertheless, a statistically significant difference was observed between Type 3 and Type 4 at *P* < 0.05. The result indicated that the effect of *qPbm9* was small.

### *Differences between* qPbm11 *and* Pb1

**Figure 6:**
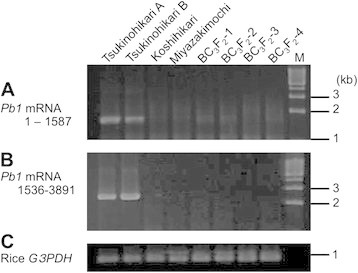
**RT-PCR pattern of*Pb1*in backcrossed progeny between Miyazakimochi and Koshihikari.** RT-PCR was used to target two subregions of the *Pb1* coding region: **(A)** from 1 to 1587 bp, and **(B)** from 1536 to 3891 bp. The primers were designed with reference to Hayashi et al. ([2010b]) and the *Pb1* genomic sequence (AB570371). Rice *G3PDH* (glyceraldehyde-3-phosphate dehydrogenase, AK064960) was used as a control gene **(C)**. RT-PCR was carried out with total RNA extracted from panicles of Tsukinohikari (two individuals, A and B), Koshihikari, Miyazakimochi, and four BC_3_F_2_ individuals (BC_3_F_2_-1 to −4). Tsukinohikari and Koshihikari were used as positive and negative controls for *Pb1* amplification, respectively. M = molecular size marker (1 kb Ladder marker, TAKARA).

Genotyping of the 24 BC_2_F_7_ individuals narrowed the location of *qPbm11* to the region between markers RM26890 and RM27207 (Figure 5B). This region overlaps with the *Pb1* locus (Hayashi et al. [2010b]). We checked the presence of *Pb1* in Miyazakimochi and four BC_3_F_2_ individuals by reverse-transcriptase polymerase chain reaction (RT-PCR). The BC_3_F_2_ individuals had Miyazakimochi-derived homozygous genotypes between markers RM206 and Miy11269; this is in the *qPbm11* region (data not shown). No *Pb1* transcript was detectable in the panicles of Miyazakimochi or its progeny (Figure 6). This indicated that *qPbm11* controlling panicle blast resistance in Miyazakimochi is a novel QTL distinct from *Pb1*.

## Discussion

Rice panicle blast is a serious disease that directly reduces the number of grain fillings, and the weight and quality of grains. Therefore, control of panicle blast is very important for stable rice production. Reducing the reproduction of rice blast on leaves leads to a reduction in the opportunity for blast infection of the panicles. Many leaf blast resistance genes, including *pi21*, *Pi34*, *Pi35,* and *Pi39* have been introgressed into rice cultivars (Fukuoka and Okuno [2001]; Zenbayashi et al. [2002]; Nguyen et al. [2006]; Terashima et al. [2008]), and these cultivars are used to control panicle blast indirectly. There are far fewer reports of genetic analyses of rice panicle blast resistance compared with leaf blast resistance. Only *Pb1*, derived from the *indica* cultivar Modan, is currently used in rice breeding in Japan. We have demonstrated that Miyazakimochi shows clear resistance to panicle blast, even under high-pressure conditions of blast disease. We identified new QTLs for panicle blast resistance through QTL analysis of progeny populations derived from a cross between the resistant Miyazakimochi and susceptible Bikei 22 *japonica* cultivars.

Frequency distributions of the panicle blast severity in the three tested generations in different fields (Figure 3A, B, and C) suggested that multiple loci are involved in panicle blast resistance of Miyazakimochi. In fact, QTL analysis of the RILs revealed the presence of two QTLs for panicle blast resistance in Miyazakimochi: *qPbm9* on chromosome 9, and *qPbm11* on chromosome 11 (Figure 3). The contribution of *qPbm11* to panicle blast resistance was 30.8%, and BC_2_F_8_ lines harboring the *qPbm11* region showed similar resistance levels to the donor parent Miyazakimochi. Thus, these results demonstrated that the resistant phenotype of Miyazakimochi is mostly attributable to the major QTL *qPbm11*. The effect of *qPbm9* on panicle blast resistance in Miyazakimochi was small and it might be difficult to detect under the coexistence of the major QTL *qPbm11* in a high-pressure field for panicle blast.

Before this study, two preliminary QTL analyses were performed at Daisen in 2001 and at Gozenyama in 2003 (La et al. [2002]; Koizumi et al. [2005]), as mentioned in the Background. Both QTL analyses indicated the presence of a major QTL on the long arm of chromosome 11, and the location of the QTL corresponded to the *qPbm11* region, suggested from the information of the marker sequences. The phenotypic data of these preliminary analyses, shown in Figure 3A and B, suggested the involvement of multiple loci in panicle blast resistance, similar to that in Figure 3C. Nevertheless, besides the major QTL, no minor QTLs were detected in the first QTL analyses in a mid-pressure field for blast disease at Daisen (La et al. [2002]). The second QTL analysis in a high-pressure field for blast disease at Gozenyama suggested a minor QTL on the end of the short arm of chromosome 11 (Koizumi et al. [2005]). In the present study, the effect of the minor QTL on panicle blast resistance was examined through analysis of a BC_2_F_7_ population lacking this minor QTL on the end of short arm of chromosome 11 (derived from MBF_6_-26). Given that Type 2 lines harboring solely *qPbm11* showed a similar level of resistance to panicle blast as Miyazakimochi (Figure 5A), the contribution of minor QTLs to blast resistance, including *qPbm9*, is suggested to be negligible or small. The experimental field for this assay was Inabu, which is a high-pressure condition for blast disease, especially panicle blast (Fujii and Hayano-Saito [2007]), in which the difference in parental disease severity was very small (Figure 3C). Thus, there was a possibility that minor QTLs might be underestimated. An assay under gentler conditions for panicle blast disease might be required to evaluate the effect of minor QTLs. However, because of the difficulty and complexity of evaluating panicle blast resistance, the QTL analyses were influenced by many factors. When considering the results, including the preliminary analyses, it is possible that the involvement of multiple loci suggested by the disease severity distribution may be a result of integrated influences from minor factors. Clarification of the contribution of each QTL to panicle blast resistance requires further research. Overall, we conclude that the major QTL *qPbm11* is responsible for resistance to panicle blast in Miyazakimochi.

Many nucleotide-binding site leucine-rich repeat (NBS-LRR) encoding genes, which are typically the major resistance (*R*) genes in plants, are not randomly scattered on all chromosomes (Zhou et al. [2004]). A recent review (Miah et al. [2013]) pointed out that 24 of the 96 known rice blast *R* genes are located on chromosome 11. As *qPbm11* is found on chromosome 11, further studies are required to clarify its relationship with other known *R* genes, and especially the panicle blast resistance gene *Pb1*, whose locus is located in the *qPbm11* region. It is reasonable to assume that *qPbm11* is distinct from *Pb1*; *Pb1* is derived from the *indica* cultivar, Modan (Fujii and Hayano-Saito [2007]), but cultivars originating from *indica* are not found in the Miyazakimochi pedigree (Uchiyamada et al. [1979]). In support of this assumption, we confirmed that *Pb1* is not expressed in panicle blast-resistant Miyazakimochi*.* Nevertheless, the possibility remains that *qPbm11* is an allele of *Pb1* or a *Pb1* family gene [e.g., Os11g0597400, Os11g0597700, Os11g0598300, and Os11g0598500 (Hayashi et al. [2010b])], located in the *qPbm11* region of Miyazakimochi. It is necessary to determine whether the allele and *Pb1* family genes function as *R* genes for panicle blast in Miyazakimochi.

Resistance to panicle blast is correlated with leaf blast in many rice cultivars (Bonman et al. [1992]). However, some rice cultivars show different levels of partial resistance to leaf and panicle blast (Bonman et al. [1992]; Shindo and Asaga [1989]). In Miyazakimochi, resistance to panicle blast shows a weak correlation with leaf blast resistance (Figure 3D, E, and F). This specificity of resistance in Miyazakimochi to panicle blast makes it a good resource for application in rice breeding programs, and facilitates the analysis of the molecular basis of panicle blast resistance.

Breeding blast resistant cultivars is considered an eco-friendly, effective and economical way to control the disease. Several strategies have been proposed to control blast using resistant cultivars, including multilines (Koizumi et al. [2004]), mixtures (Mundt [2002]), and gene pyramiding (Fukuoka et al. [2012]). Leaf blast resistance is mainly used to control panicle blast indirectly at present. New panicle blast resistances will be utilized in protection strategies against blast disease, together with leaf blast resistance. Furthermore, for strategies using the host’s *R* genes, the key is furthering rice breeding efficiently. Genomic sequence information and the richness of markers linked to agronomic traits has permitted marker-associated-selection to be applied generally in rice breeding programs in Japan, especially for imparting disease resistance and pest tolerance. Agronomical target traits are expected to be introgressed efficiently by DNA markers, without any accompanying poor traits. Furthermore, accumulating research on rice *R* genes and their functions will lead to the breeding of resistant rice cultivars based on the functional mechanism of the introduced *R* gene(s).

## Conclusions

This study used three experimental fields under different pressure from rice blast to demonstrate that the rice cultivar Miyazakimochi harbors panicle blast resistance without correlation with leaf blast resistance. QTL analysis using RILs derived from a cross between Miyazakimochi and Bikei 22 indicated that two QTLs, *qPbm9* and *qPbm11,* were involved in panicle blast resistance. Analysis of homozygous backcrossed lines in the QTL regions revealed that *qPbm11* is predominantly responsible for panicle blast resistance in Miyazakimochi. The *qPbm11* region contains a locus for the panicle blast resistance gene *Pb1* (Hayashi et al. [2010b]); however, RT-PCR confirmed that *Pb1* was not expressed in Miyazakimochi. *qPbm11* is a novel genetic source for the breeding of panicle blast resistance in rice. *qPbm11* will provide an additional target for varietal improvement of panicle blast resistance through marker-associated-selection, and represents an important genetic resource for further understanding of panicle blast resistance.

## Methods

### Plant materials

Miyazakimochi is a *japonica* rice cultivar that has resistance to panicle blast and partial resistance to leaf blast. Bikei 22 is a susceptible *japonica* rice cultivar (Figure 1), with a similar heading date to Miyazakimochi. We developed an F_2_ population from a cross between Miyazakimochi and Bikei 22 (Figure 2), and F_3_ lines were used to characterize the panicle resistance of Miyazakimochi in Daisen. One hundred seventy-one F_6_ RILs from the same population were generated using a single-seed descent method (Figure 2). The 171 F_6_ RILs were used for assays of rice blast in Gozenyama. Following these analyses, seeds of 13 lines of the 171 F_6_ RILs were used up, and these lines were lost. The remaining 158 F_6_ RILs and their F_7_ generations were used for genotyping and evaluation of blast resistance QTL analysis, respectively, in Inabu.

To investigate the contribution of the detected QTLs, we developed a derivative population, MBF_6_-26/2*B, in which Bikei 22 was backcrossed twice to resistant MBF_6_-26 (Figure 2). MBF_6_-26 had Miyazakimochi-derived homozygous regions encompassing the QTLs detected by the QTL analysis. The BC_2_F_7_ individuals and the BC_2_F_8_ lines were used for genotyping and evaluation of panicle blast resistance, respectively.

For RT-PCR of *Pb1* expression, we used Miyazakimochi and four BC_3_F_2_ individuals where the *japonica* susceptible cultivar Koshihikari was backcrossed four times to Miyazakimochi. The cultivar Tsukinohikari, which harbored *Pb1* derived from Modan, was used as a positive control, and the cultivar Koshihikari was used as a negative control.

### Evaluation of field resistance to blast

Field assays in three different experimental paddy fields, and one upland nursery field in Daisen, Gozenyama, and Inabu were used to evaluate blast resistance (Figure 2). The Daisen fields in Akita prefecture possess suitable field conditions for blast disease development (Shindo and Asaga [1989]), and were used in 2000 for evaluation of panicle blast resistance in an experimental paddy field, and leaf blast resistance in an upland nursery field using F_3_ lines. The Gozenyama experimental field is a high-pressure field for blast disease in Ibaraki Prefecture, and was used to evaluate resistance to panicle blast and leaf blast in F_6_ RILs in 2003. The Inabu field is also a high-pressure field for blast disease, especially panicle blast (Fujii and Hayano-Saito [2007]), and is located at the Mountainous Region Agricultural Research Institute of the Aichi Agricultural Research Center; it was used for assays of F_7_ RILs in 2011, and BC_2_F_8_ lines in 2012 and 2013. Data assayed in the three fields were used to characterize panicle blast resistance of Miyazakimochi, and any correlations to leaf blast resistance. The Spearman rank correlation was used to assess correlations between the scores of panicle blast and leaf blast severities.

Field assays were performed under natural conditions as follows. In the paddy field trials, 15 plants of each F_3_ line, RIL, BC_2_F_8_ line, and the parental cultivars were transplanted by hand, with one plant per hill in a 270, 225, and 90 cm row at a distance of 18, 15, and 6 cm in Daisen, Gozenyama, and Inabu, respectively. Fifteen plants from the respective parental cultivars were transplanted alternately for comparison in every second (Daisen), or fifth (Gozenyama and Inabu) row. The rows were spaced at 24.5 cm in Daisen, and 30 cm in Gozenyama and Inabu. A completely randomized block design was used, with three replications. Leaf blast severity was evaluated by visual assessment of the highest stage of disease development in each row. Resistance evaluation was scored from 0 (no lesion) to 10 (all plants killed by the disease) according to the disease severity index developed by Asaga ([1981]). Panicle blast severity was evaluated in each row five times, every 2 or 3 days after the heading date. This evaluation was based on visual assessment of disease severity, as described by Asaga ([1981]); scores ranged from 0 (no diseased grain) to 10 (100% diseased grain). Phenotypic data, as measured by panicle blast severity scores, were used when the largest difference was seen between the two parental rice cultivars.

In the upland nursery trial in Daisen, 50 seeds from each F_3_ line and the parental cultivars were sown in a 40 cm length row with 10 cm spacing. The F_3_ lines were planted in alternate rows, with the parental cultivars between them. A completely randomized block design was used, with two replications. To induce leaf blast development, a rice *japonica* susceptible cultivar was planted as a spreader in 10 rows at both sides of each block, and blast-diseased leaves were scattered on the experimental plots. The evaluation of leaf blast severity in each of the F_3_ lines and in the parental cultivars was performed as described above.

### DNA markers

Thirty-five SSR markers [prefixed RM## (McCouch et al. [2002]; International Rice Genome Sequence Project [2005], IRGSP) and HvSSR## (Singh et al. [2010])] that showed polymorphisms between Miyazakimochi and Bikei 22 were selected. Investigation of single nucleotide polymorphism (SNP) loci between Miyazakimochi and Bikei 22 was based on loci between Nipponbare and Koshihikari (Nagasaki et al. [2010]). From these SNPs, 105 SNP markers, six cleaved amplified polymorphic sequence markers (prefixed aa##), and one sequence tagged site marker (STS1) were developed (Additional file 1). The method described by Hayashi et al. ([2004]) was used to design the primers for the SNP markers. The 3'-end of one of the primer pairs was specific to the SNP site, with the third position from the 3'-end having artificially introduced mismatch bases to reduce false-positive amplification. In addition to the above, Pb3810/16, which is linked to the panicle blast resistance gene *Pb1* (Hayashi et al. [2010b]), was also used. In total, 147 markers were used for QTL analysis of the RILs.

Fifteen co-dominant markers were used for genotyping of the MBF_6_-26/2*B population. The two markers RM6491 and RM1553 (IRGSP 2005) are located on chromosome 9, while the other 13 markers are on chromosome 11. These included previously developed markers aa11006712, aa11001573, nksrssr11_19358 (Singh et al. [2010]), RM206, RM26890, RM27147, RM27185, RM27207 (IRGSP 2005) and Pb3810/16; and four newly developed markers, Miy11256, 5083indel, Miy11269 and Miy11276 (Additional file 1). Sequence data for Miyazakimochi and Bikei 22 were used for marker development. Paired-end sequencing using an Illumina Hiseq 2000 apparatus (Illumina Inc. San Diego, CA, USA) was used to analyze the whole genome sequences of Miyazakimochi and Bikei 22, and DNAnexus (DNAnexus Inc., Mountain View, CA, USA; https://dnanexus.com) was use to compare the sequence data with the Nipponbare genome sequence (build 5, http://rapdblegacy.dna.affrc.go.jp/download/index.html) as a reference. Hokkaido System Science Co., Ltd. (Sapporo, Hokkaido, Japan) carried out these operations.

### Genotyping analysis

The cetyl trimethyl ammonium bromide method (Murray and Thompson [1980]) was used to extract total rice DNA from each plant. PCR with SNP markers used a 10 μl reaction mixture containing 50 ng of template DNA, 0.125 μM of each primer, 0.25 U of *Taq* HS (Takara, Ohtsu, Japan), 0.4 mM of each dNTP, and 1 × PCR buffer. PCR with other markers used a 10 μl reaction mixture containing 50 ng of template DNA, 0.2 μM of each primer, 5 μl of 2 × GoTaq green master mix (Promega, Madison, WI, USA). Amplification used 28 to 45 cycles at annealing temperatures of 50 to 68°C, depending on the primers. Amplified DNA fragments were separated on 3% (w/v) agarose gels, and visualized using ethidium bromide staining.

### QTL analysis

MAPMAKER/EXP (version 3.0b; Lincoln et al. [1993]), set to the Kosambi mapping function in cM, was used to construct a genetic linkage map from the segregating data of the RILs. An individual linkage group was declared if any distance between two adjacent markers was less than 100 cM. A LOD threshold of 3.0 constructed an initial framework, and remaining markers added to the framework with the LOD threshold set at 2.0 or 1.0. For QTL analysis, composite interval mapping (CIM) was performed using Windows QTL Cartographer software (version 2.5; Wang et al. [2010]) to determine the genomic regions (marker intervals) associated with panicle blast resistance. The software parameters were set as follows: a standard Model 6 with a control markers number of 5, a window size of 10 cM around the test interval, a walk speed of 1 cM, and ‘backward regression method’ as cofactors. The empirical LOD threshold for the trait was determined by 1,000 permutations at the *P* < 0.05 level (Churchill and Doerge [1994]). CIM analyses provided estimates of additive effects and the percentage of phenotypic variance explained by the putative QTL.

### RT-PCR

TRIzol reagent (Invitrogen, Carlsbad, CA, USA) was used to extract total RNA from panicles of Tsukinohikari, Koshihikari, Miyazakimochi, and four BC_3_F_2_ individuals, according to the manufacturer’s instructions. For expression analysis, RT-PCR was carried out using an RNA-PCR kit (Takara) with 0.5 μg of total RNA in each sample. RT-PCR targeted two subregions of the *Pb1* coding regions: from 1 to 1587 bp, and from 1536 to 3891 bp. Primers were designed with reference to the supplemental data of Hayashi et al. ([2010b]) and the genomic sequence of *Pb1* (Accession No. AB570371). The primer sequences were: 5'-ATGACTGAGCTCGCGTCTGG-3' and 5'-ATCGGGTCTTCATCATCATCATC-3' for the 1–1587 subregion, and 5'-TGGTGAAGAAGAAGAAGAAGAAGA-3' and 5'-TGGTTCATTACATTTAAGAATTATCC-3' for the 1536–3891 subregion. Rice *G3PDH* (glyceraldehyde-3-phosphate dehydrogenase, Accession No. AK064960) was used as a control gene to standardize the RT-PCR results. The primer sequences were: 5'-ACAACTGTTCATGCCATCAC-3' and 5'-TCGATGACACGGTTGCTGTA-3'. RT-PCR products were subjected to electrophoresis on a 1% (w/v) agarose gel, and detected by ethidium bromide staining.

## Competing interests

The authors declare that they have no competing interests.

## Authors’ contributions

TI contributed to the experimental process, data analysis, and manuscript preparation. SO contributed to the genotyping and QTL analysis. KE contributed to the detection of SNP sites and marker design. NTL contributed to the Gozenyama phenotyping. KH contributed to the genetic analysis, results interpretation and manuscript preparation. TA and FS contributed to the phenotyping and manuscript preparation. YH-S contributed to the genotyping, data analysis, results interpretation and wrote the manuscript. SK contributed to the planning of this study, coordination of experimental processes of phenotyping and all data analysis. All authors have read and approved the manuscript.

## Additional file

## Supplementary Material

Additional file 1 Primer sequences of markers developed for the genetic analysis. Click here for file
